# Perceptions of medical doctors on bisphosphonate-related osteonecrosis of the jaw

**DOI:** 10.1186/s12903-016-0290-0

**Published:** 2016-09-07

**Authors:** Jin-Woo Kim, Su-Ra Jeong, Sun-Jong Kim, YeonSoo Kim

**Affiliations:** 1 Graduate School of Clinical Dentistry, Ewha Womans University, Seoul, Korea; Research Institute for Intractable Osteonecrosis of the Jaw, School of Medicine, Ewha Womans University, Seoul, South Korea; 2 Department of Medical & Pharmaceutical Science, Sookmyung Women’s University, Seoul, South Korea; 3 Department of Oral & Maxillofacial Surgery, Ewha Womans University Medical Center, Anyangcheon-ro 1071, Yangcheon-gu, Seoul 158-710 South Korea

**Keywords:** Osteonecrosis of the Jaw, Bisphosphonate, Awareness, Questionnaire, Doctor

## Abstract

**Background:**

This study aimed to investigate medical doctors’ awareness of bisphosphonate-related osteonecrosis of the jaw (BRONJ) and the status of dental referrals.

**Methods:**

Self-administered questionnaires were distributed to medical doctors practicing internal medicine, family medicine, and orthopedics at the 6 tertiary medical centers located in Seoul, Korea. The survey consisted of 22 questions regarding the general characteristics, bisphosphonate therapy, awareness of BRONJ, and implementation of dental referrals.

**Results:**

Among 192 medical doctors, 21.9 % had not heard of the disease. Only 8.9 % correctly answered all 5 questions testing BRONJ knowledge. Dental referrals made by medical doctors were implemented in less than 30 % of the total patients. Oncology specialists most often recognized the necessity of dental referrals followed in decreasing order by endocrinology, rheumatology, family medicine, and orthopedic specialists.

**Conclusion:**

Given medical doctors’ low BRONJ perception and implementation level of dental referrals, enhancing information dissemination on BRONJ and development of a highly accessible educational program recognizing the need for dental referrals are urgent.

**Electronic supplementary material:**

The online version of this article (doi:10.1186/s12903-016-0290-0) contains supplementary material, which is available to authorized users.

## Background

Bisphosphonates (BP) are a class of drugs used in the treatment and prevention of various bone diseases, such as osteoporosis, osteitis deformans, multiple myeloma, osteogenesis imperfecta, and bone metastases, by suppressing osteoclast-mediated bone resorption through apoptosis of osteoclasts [1]. Due to the drug’s safety and strong bone-specific inhibitory effect on osteoclastic activity, BPs have effectively been used for decades [2, 3]. However, in the recent years, there has been a serious adverse effect of jaw necrosis associated with the administration of BPs [4, 5]. Although the pathophysiology of jaw necrosis due to drug use is not yet been fully understood, significant inhibition of bone remodeling based on the drug’s pharmacological effect has been considered to be the main cause [6–8].

After the first report of bisphosphonate-related osteonecrosis of the jaw (BRONJ) in 2003 [4], the American Association of Oral and Maxillofacial Surgeons (AAOMS) defined BRONJ in their 2009 position paper as “necrotic bone exposure in the maxillofacial region lasting for more than 8 weeks in patients with previous or current administration of BP and with no history of radiation therapy” [9]. In the latest position paper in 2014, AAOMS modified the term into medication-related osteonecrosis of the jaw (MRONJ) to emphasize the role of drugs causing osteonecrosis of the jaw [7]. The incidence of BRONJ has been reported to be 0.01 to 6.7 % [7]. Considering the worldwide trend of increasing BP therapy and high BP dependence, the incidence is expected to continuously grow [10]. Although the treatment modalities are not established, most researchers have recommended conservative approaches and the additional use of growth factors or hormone therapy [7, 11].

In 2009, Park et al. [12] reported that only 56.5 % of Korean dentists were aware of BRONJ, and another study reported similar awareness in dental hygienists [13]. Therefore, efforts to increase the awareness of BRONJ among oral health professionals have been made, emphasizing the importance of prevention of the disease and research on intractable osteonecrosis of the jaw.

However, there have been few studies on medical doctors’ awareness of BRONJ and how well dental referrals are being carried out. As in other forms of osteonecrosis, such as osteoradionecrosis and osteomyelitis of jaw, BRONJ usually has a poor prognosis and demands long-term treatment, thus mutual efforts between the medical doctor and dentist through a multi-disciplinary approach are essential in the diagnosis, treatment, and prevention of BRONJ [14]. Therefore, this study aimed to investigate the awareness of BRONJ among medical doctors who prescribe BPs and the current situation of dental referrals in the multi-disciplinary treatment and prevention of BRONJ.

## Methods

Among 17 tertiary medical institutions in Seoul, Korea which are registered with the Health Insurance Review Agency as of July of 2015, 6 were selected based on a random sampling method. The subjects from the selected institutions were medical specialists and residents of internal medicine (endocrinology, rheumatology, oncology), family medicine, and orthopedics who agreed to participate in the study.

The number of subjects was determined to be 172 based on Cohen’s sample size calculations with a power of more than 90 % and a significance level of 0.05 (two-sided test). After accounting for dropouts from the study, a total of 200 subjects were used [15]. The study was carried out by visiting the participating institutions and distributing self-administered questionnaires. After thoroughly explaining the purpose and content of the study, the surveys were given out to only those who voluntarily chose to participate in the study. Surveys that were not collected on the same day of distribution were collected during another visit to the institutions or were sent through mail. Of the 200 surveys, 192 were used in the analysis after excluding 4 uncollected surveys and 4 surveys with insufficient answers. This study was approved by the Ethics Committee of Ewha Womans University, Seoul, Korea (15-16B-16).

In the study, the terminology ‘BRONJ’ rather than ‘MRONJ’ or ‘ARONJ’ (antiresorptive-related osteonecrosis of the jaw) was used. Considering that the target sample of this survey was medical doctors who do not treat ONJ themselves and are unfamiliar with the relatively novel terminology MRONJ, the term BRONJ was preferentially used. Also, because antiresorptive agents include not only BP and monoclonal antibodies, but also calcitonin, estrogens, and selective estrogen receptor modulators, the authors considered the terminology BRONJ rather than ARONJ to be more appropriate for this study.

The survey was comprised of a total of 22 questions. Personal information was not included. First, the surveys were categorized according to the specialties: internal medicine (endocrinology, rheumatology, oncology), family medicine, and orthopedics. Period of career was divided into the following: (1) less than 5 years, (2) between 5 and 10 years, (3) between 10 and 20 years, and (4) more than 20 years. Questions regarding BP treatment, the reason for administration, the doctors’ level of awareness, and dental examination and referrals were also included in the questionnaire. (See Additional file 1) The medical doctors’ understanding of BRONJ was evaluated using the AAOMS guidelines, consisting of questions regarding the definition, staging, treatment, and prevention of the disease, and the discontinuation of BP [7].

Descriptive statistics were utilized for the general characteristics of samples, BP therapy, experience with BRONJ patients, and the doctors’ sources on BRONJ information. For evaluating the degree of understanding, 1 point was given for each correct answer to the questions regarding the definition, staging, treatment, prevention, and drug discharge. No points were given when the subject answered incorrectly or by answering ‘I do not know.’ Higher points correspond to higher level of understanding. The group differences in level of understanding were evaluated according to job characteristics, BP indication, administration route, and experience of BRONJ patients.

For evaluating the degree of dental referrals, the question was based on a 5-point scale with answer choices of ‘before’, ‘during’, and ‘after BP administration’. When 100 % of patients requiring BP administration were being referred to dentists, 5 points were given, and on the other hand, when less than 10 % were being referred, 1 point was given. The reasons for not referring patients to dentists were presented, and the group differences of dental referrals were evaluated according to job characteristics, BP indication, administration route, doctor’s awareness, and experience with BRONJ patients.

Statistical analyses were performed using SPSS 20.0 (SPSS Inc., Chicago, IL, USA). Frequency and percentage analysis, chi-square test, independent sample T-test, and one-way analysis of variance were performed. For post hoc testing, Scheffe’s test was used. All values were considered statistically significant when *P* < 0.05.

## Results

General characteristics of the 192 surveyed medical doctors, BP therapy, and experience with BRONJ patients are summarized in Table 1. In the question regarding awareness of BRONJ, 150 doctors (78.1 %) answered “heard of the disease,” and 42 (21.9 %) answered “have not heard of the disease.” Medical specialists were more aware of BRONJ compared to residents (*P* < 0.01), and the highest level of awareness was seen in the group with more than 20 years of work experience, followed by less than 5 years, between 10 and 20 years, and between 5 and 10 years (*P* < 0.01). On the other hand, there was no significant difference between the areas of specialty. The majority of participants (38 %) responded that the source from which they acquired awareness of the disease was academic journals, followed by educational seminars (30 %), books (20 %), newspapers (5 %), internet (4 %), and others, such as from colleagues, or patient experiences (3 %).

**Table 1:** General characteristics of surveyed medical doctors, bisphosphonate (BP) therapy and experience of bisphosphonate-related osteonecrosis of the jaw (BRONJ) patients (*n* = 192)

	Group	*N*	Percent
Position	Specialists	114	59.4
	Residents	78	40.6
Specialty	Family medicine	50	26.1
	Orthopedics	55	28.6
	Internal Medicine	87	45.3
Period of Career	<5 years	69	35.9
	5 ~ 10 years	33	17.2
	10 ~ 20 years	36	18.8
	>20 years	54	28.1
Indications for BP use	Osteoporosis	160	83.3
	Cancer	31	16.2
	Others	1	0.5
Administration route of BP	Intravenous	63	32.8
	Oral	127	66.2
	Other	2	1.0
Other adjuvant medications used with BP	Corticosteroids	20	10.4
	Chemotherapy	22	11.5
	None	150	78.1
Number of patients on BP prescription per month	1–10	111	57.8
	11–20	26	13.5
	Over 21	55	28.7
Experience of patients with BRONJ	Yes	53	27.6
	No	115	59.9
	Do not know	24	12.5
Number of patients with BRONJ (*n* = 53)	1–3	35	66.0
	3–6	16	30.2
	Over 6	2	3.8

To determine the level of BRONJ understanding, 5 questions concerning the definition, staging, drug discontinuation, prevention, and proper management of the disease were included. Only 17 doctors (8.9 %) answered all 5 questions correctly, and 20.9 % (*n* = 21) received zero points. Medical specialists had higher scores compared to residents, and the group with 10 to 20 years of work experience had the highest level of BRONJ knowledge (*P* < 0.01). Also, medical doctors who had experience with BRONJ patients showed a significantly higher awareness score.(*P* < 0.01). However, there was no statistically significant difference in the level of BRONJ knowledge between the areas of specialty (*P* > 0.05; Table 2).

**Table 2:** Assessment of Knowledge on bisphosphonate-related osteonecrosis of the jaw (BRONJ)

		BRONJ Knowledge		*P* value
		*N*	M ± SD	
Position	Specialist	78	1.99 ± 1.392	.001
	Resident	114	2.66 ± 1.314	
Period of Career	<5 years	69	1.88 ± 1.40	.002
	5 ~ 10 years	33	2.45 ± 1.23	
	10 ~ 20 years	36	2.83 ± 1.38	
	>20 years	54	2.63 ± 1.31	
Specialty	Family medicine	50	2.52 ± 1.58	.106
	Orthopedics	55	2.04 ± 1.32	
	Internal medicine	87	2.37 ± 1.39	
Experience of BRONJ patients	Yes	53	3.04 ± 1.34	<0.001
	No	139	2.14 ± 1.49	
Indications for use (*n* = 191)	Osteoporosis	160	2.26 ± 1.319	.009
	Cancer	31	2.97 ± 1.602	

*M* mean score of total correct answers on BRONJ

According to the 5-point scale, the degree of dental referrals for BP administration was 1.86 ± 1.2 before administration, 1.99 ± 1.2 during administration, and 1.78 ± 1.1 after administration. The results were not significantly different according to the participants’ position and period of career. On the other hand, based on the areas of specialty, before BP administration (*P* < 0.01), during administration (*P* < 0.01), and after administration (*P* < 0.05), all displayed statistically significant differences with internal medicine showing the highest degree of dental referrals, followed by family medicine and orthopedics. Also, the group with BRONJ awareness and prescribing BP to prevent bone metastases showed a higher degree of dental referrals (*P* < 0.01) compared to the group prescribing the drug for treatment of osteoporosis.

**Fig. 1:**
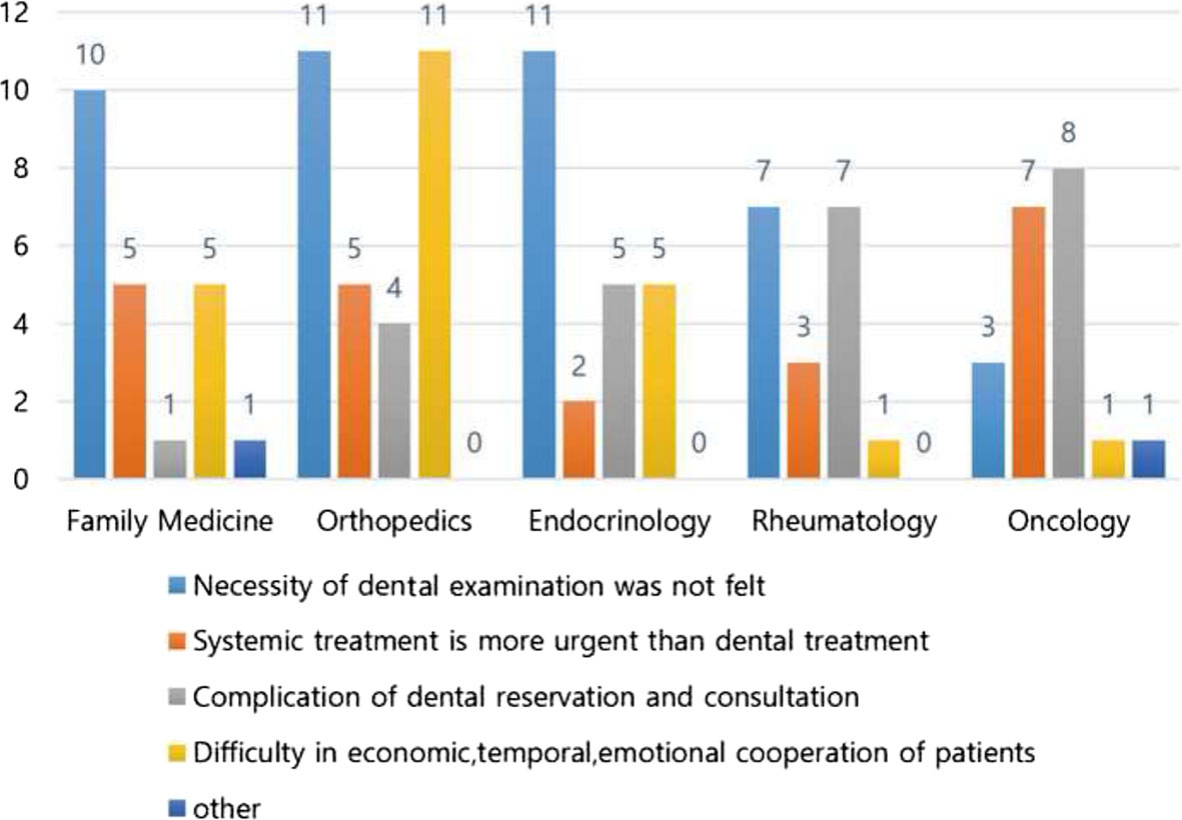
Reasons why medical doctors do not make dental referrals

The perception of the necessity of dental referrals according to the areas of specialty was highest in oncology (4.30 ± 1.08), followed by endocrinology (3.65 ± 0.98), rheumatology (3.44 ± 1.42), family medicine (3.36 ± 1.22), and orthopedics (3.12 ± 1.21) (*P* < .05). However, the degree of dental referrals did not show a statistically significant difference among the areas of specialty (*P* > 0.05). The reasons for not making dental referrals differed among the specialties (Fig. 1). In family medicine, endocrinology, and rheumatology, the majority answered that they did not feel the necessity of dental referrals; in orthopedics, it was difficult to earn patient cooperation due to financial, time, and motivational issues; and in oncology, they thought that the treatment of systemic disease was more urgent than dental treatment.

## Discussion

Since the first report of BRONJ in 2003, there have been various clinical and experimental studies, yet much of the treatment and prognosis of the disease remain unknown [16]. Considering the refractory progress of BRONJ, it has widely been accepted that the best measure for BRONJ treatment is prevention. Therefore it is critical for medical doctors to implement dental referrals for pre-evaluation of oral health and dental risk factors before administering BP [7, 14, 17].

In 2005, the FDA attempted to increase BRONJ awareness among healthcare providers by adding a warning statement of possible osteonecrosis of the jaw through administration of BP. However, in this study, 21.9 % of the medical doctors surveyed had not heard of the disease, and only 9.9 % correctly answered all 5 questions, indicating a very limited BRONJ awareness.

The knowledge score was significantly higher in doctors who had experience with BRONJ patients. This could be due to their efforts to obtain more information relevant to the cases that they have handled. As a result, the need for educating those who showed lower scores is paramount particularly if they have not handled actual cases. Encountering the development of ONJ before proper education and through understanding of the disease could lead to poor medical practice. Considering that knowledge of BRONJ was acquired mainly through academic journals (38.5 %) and educational seminars (30.3 %), continuous educational publications and development of highly accessible educational programs for medical doctors are deemed urgent and necessary.

Kholsa et al. [11] and Lam et al. [17] reported that management of periodontal disease and oral hygiene are most important in patients who require treatment with BP, thus emphasizing the necessity of informing them of the risk of BRONJ and importance of performing dental treatment before and during drug administration. However, according to the present study, the percentage of dental referrals before, during, and after the administration of BP remains low, in less than 30 % of total patients. Given the reasons for not making dental referrals according to the area of specialty as stated previously, the need to express the extreme importance for dental examination and generate a well-coordinated referral system is recommended through open communication and exchange of expertise from both fields.

Several studies recommended that thorough history taking, mutual exchange between the dentist and medical specialist, and providing detailed information of BRONJ to patients are essential to its prevention [14, 16]. For the prevention of BRONJ, unsalvageable teeth are recommended to be extracted before starting BP treatment [7, 14]. Also, if all dental procedures are performed prior to BP administration, future invasive procedures such as dental extractions can be prevented. Preventive measures for optimal oral health can lead to a decrease in the number of BRONJ patients [7].

A limitation of this study is that the subjects were 192 medical doctors selected through random sampling in tertiary hospitals, therefore the authors cannot declare with certainty that the sample represents all medical doctors nor can the authors exclude selection bias. Further studies with a sufficient sample size and joint research with the other medical societies would provide more reliable results.

## Conclusion

Through this study, it was shown that the medical doctors’ awareness of BRONJ was low. Based on the finding that only 30 % of patients taking BPs are referred to dentists, these results are valuable as basic educational data to not only express the urgency and severity of BRONJ to medical doctors but to emphasize the utmost importance of prevention through mutual communication between dentists and medical doctors as well.

## Additional file

Additional file 1: Questionnaire. (DOCX 30 kb)

## Abbreviations

AAOMS: American Association of Oral and Maxillofacial Surgeons
BP: Bisphosphonates
BRONJ: Bisphosphonate-related osteonecrosis of the jaw
FDA: Food and Drug Administration
MRONJ: Medication-related osteonecrosis of the jaw
