# Supplementary material for: Perceptions of medical doctors on bisphosphonate-related osteonecrosis of the jaw
Source: BMC Oral Health. 2016 Sep 7;16(1):92. doi: 10.1186/s12903-016-0290-0 (PMC5015333; doi:10.1186/s12903-016-0290-0)
Supplement: Additional file 1: — Questionnaire. (DOCX 30 kb) [file 12903_2016_290_MOESM1_ESM.docx]

**Questionnaire**

| **The following questions are on general characteristics.**  **Check the corresponding number or directly write the answer.** |
| --- |

1. What is your job position at the hospital?

1) Resident 2) Specialist

2. How much work experience do you have (after obtaining dental license)?

( ) years ( ) months

3. What is your area of specialty?

1) Internal medicine

(Please choose your subspecialty**:** ①Endocrinology ②Oncology ③Rheumatology )

2) Orthopedics

3) Family medicine

| **The following questions are on bisphosphonate treatment.**  **Check the corresponding number or directly write the answer.** |
| --- |

1. On average, to how many patients do you prescribe bisphosphonates per month?

( ) patients per month

2. What is the main reason for prescribing bisphosphonates?

1) Osteoporosis 2) Cancer 3) Etcetera ( )

3. Which form of bisphosphonates do you prescribe the most?

1) Intravenous 2) Oral 3) Others ( )

4. What adjunctive therapy do you use with bisphosphonates?

1) Corticosteroids 2) Chemotherapy 3) None

| **The following questions are on the awareness of BRONJ.**  **Check the corresponding number or directly write the answer.** |
| --- |

1. Have you heard of BRONJ (bisphosphonate-related osteonecrosis of the jaw)?

1) Yes 2) No → (Go to question 2)

1-1. If yes, how did you hear about it? (multiple answers are possible)

1) Internet 2) Newspaper 3) Academic journals 4) Books 5) Lectures/seminars

6) Others ( )

2. Do you consider yourself to have a good understanding of BRONJ?

| To a large extent | To a moderate extent | To some extent | To a small extent | Not at all |
| --- | --- | --- | --- | --- |
|  |  |  |  |  |

3. Have you experienced any patients with BRONJ?

1)Yes → (Go to question 3-1) 2) No 3) Do not know

| **The following questions are on dental referrals.**  **Check the corresponding number or directly write the answer.** |
| --- |

3-1. How many patients with BRONJ symptoms have you experienced? ( ) patients

| **• Always**: Perform on all patients (100%)  **• Often**: Perform depending on the situation, 80% of patients  **• Sometimes:** Perform depending on the situation, 50% of patients  **• Seldom:** Perform depending on the situation, 30% of patients  **• Rarely:** Perform depending on the situation, less than 10% of patients |
| --- |

1. Do you think dental examinations are necessary for patients being treated with

bisphosphonates?

| Always | Often | Sometimes | Rarely | Never |
| --- | --- | --- | --- | --- |
|  |  |  |  |  |

2-4. Refer to the definitions of the answer choices above then check the corresponding

box for each question.

| Questions | Always | Often | Sometimes | Seldom | Rarely |
| --- | --- | --- | --- | --- | --- |
| 2. Do you refer patients to dentists to check on their oral health status before bisphosphonate treatment (administration)? |  |  |  |  |  |
| 3. Do you recommend regular dental check-ups to your patients while they are undergoing bisphosphonate treatment (administration)? |  |  |  |  |  |
| 4. Do you refer patients to dentists for dental examinations after bisphosphonate treatment (administration)? |  |  |  |  |  |

5. What is your reason for not referring all your patients (100%) to the dentist before, during, and after bisphosphonate usage?

1) Did not feel the necessity of dental examination

2) Systemic treatment is more urgent than dental treatment

3) Difficulty in arranging dental appointments and consultations

4) Lack of patient cooperation due to financial, time, and motivational issues

5) Others ( )

| **The following questions are on BRONJ.**  **Check the corresponding number or directly write the answer.** |
| --- |

1. Have you heard of the BRONJ Guidelines (AAOMS)?

1) Yes 2)No

2. Read the following questions and check the corresponding box.

| Questions | Yes | No | Do not know |
| --- | --- | --- | --- |
| Diagnosis of BRONJ can be made when the patient has taken bisphosphonates in the past or is currently taking them without history of radiotherapy and shows bone exposure for more than 8 weeks. |  |  |  |
| According to AAOMS, Stage 3 of BRONJ refers to exposure of necrotic bone with signs of pain and infection. |  |  |  |
| In the case of a patient with non-symptomatic BRONJ undergoing IV bisphosphonate treatment, dental extraction and implant placement are not contraindicated without any interventions such as drug discontinuation. |  |  |  |
| For patients who need to begin IV-bisphosphonates for chemotherapy, any teeth with poor prognosis must be extracted before bisphosphonate administration. |  |  |  |
| For a patient with good systemic health who has been taking oral bisphosphonates for more than 4 years, drug discontinuation is necessary for any intraoral surgical treatments. |  |  |  |
